# The Role of Mucosal Associated Invariant T Cells in Antimicrobial Immunity

**DOI:** 10.3389/fimmu.2015.00344

**Published:** 2015-07-06

**Authors:** Ruth J. Napier, Erin J. Adams, Marielle C. Gold, David M. Lewinsohn

**Affiliations:** ^1^Pulmonary and Critical Care Medicine, Oregon Health & Science University, Portland, OR, USA; ^2^The University of Chicago, Chicago, IL, USA; ^3^VA Portland Health Care System (VAPORHCS), Portland, OR, USA; ^4^Molecular Microbiology and Immunology, Oregon Health & Science University, Portland, OR, USA

**Keywords:** MR1, mucosal associated invariant T cells, antimicrobial, immunity

## Abstract

Mucosal associated invariant T (MAIT) cells are an innate-like T cell subset prevalent in humans and distributed throughout the blood and mucosal sites. Human MAIT cells are defined by the expression of the semi-invariant TCRα chain *TRAV1-2/TRAJ12/20/33* and are restricted by the non-polymorphic major histocompatibility complex (MHC) class I-like molecule, MHC-related protein 1, MR1. MAIT cells are activated by small organic molecules, derived from the riboflavin biosynthesis pathway of bacteria and fungi, presented by MR1. Traditionally, MAIT cells were thought to recognize a limited number of antigens due to usage of an invariant TCRα chain and restriction by a non-polymorphic MHC molecule. However, recent studies demonstrate that the TCR repertoire of MAIT cells is more heterogeneous, suggesting there is a more diverse array of MR1 antigens that MAIT cells can recognize. In response to infected cells, MAIT cells produce the pro-inflammatory cytokines, IFN-γ and TNF, and are cytolytic. Studies performed in MR1-deficient mice suggest that MAIT cells can provide anti-bacterial control within the first few days post-infection, as well as contribute to enhanced adaptive immunity in murine models of respiratory infections. In humans, the role of MAIT cells is unclear; however, evidence points to interplay between MAIT cells and microbial infections, including *Mycobacterium tuberculosis*. Given that MAIT cells are pro-inflammatory, serve in early control of bacterial infections, and appear enriched at tissue sites where microbes interface and gain access to the body, we postulate that they play an important role in antimicrobial immune responses. In this review, we discuss the most recent studies on the function and phenotype of MAIT cells, including their TCR diversity and antigenic repertoire, with a focus on the contribution of human MAIT cells in the immune response to microbial infection.

## Introduction

The immune system is conceptually divided into two general categories: innate and adaptive. Innate immunity is considered the first line of defense and is mediated largely by epithelial cells and phagocytes that detect and kill foreign microbes through many mechanisms, including the use of germ-line encoded receptors, referred to as pattern recognition receptors (PRRs). PRRs allow innate immune cells to quickly detect and respond to infections by binding to molecules associated with pathogens, called pathogen associated molecular patterns (PAMPs). Adaptive immunity consists of T and B lymphocytes that use rearranged antigen recognition receptors to detect a wide range of antigens. The antigen recognition receptor on T cells, the T cell receptor (TCR), recognizes foreign antigens only when they are bound to major histocompatibility complex (MHC) molecules, which are expressed on the cell surface of host cells. In general, activation through the TCR results in clonal expansion of T cells specific for a particular antigen, acquisition of effector functions, and the development of long-lived T cells, which provide immunological memory, resulting in long-term protection against subsequent re-infection.

Mucosal associated invariant T (MAIT) cells, a subset of non-classically restricted T cells, share characteristics of classical MHC-I- and MHC-II-restricted T cells, yet have unique properties that have lead to their description as “innate-like.” Like conventional T cells, MAIT cells undergo TCR rearrangement and positive selection in the thymus (1–3). However, unlike conventional T cells that remain naïve until antigenic-stimulation in the periphery, MAIT cells gain effector capacity prior to exiting the thymus (4, 5). Thus, MAIT cells have an inherent capacity to detect and respond to infection. In addition to the acquisition of innate-like features in the thymus, MAIT cells can expand and adapt following egress into the circulation (5).

In stark contrast to conventional TCRαβ T cells that recognize peptide antigens presented by MHC-I or MHC-II molecules, MAIT cells become activated by small molecules presented by the non-polymorphic MHC class I-related molecule, MR1(6). MAIT cells function similarly to conventional CD8^+^ effector T cells by secreting pro-inflammatory cytokines and cytotoxic molecules in response to microbial infections *ex vivo* (7, 8). Moreover, MAIT cells have been shown to play a role in host antibacterial responses *in vivo* (9–11). In this review, we will present compelling evidence suggesting MAIT cells serve as sentinels of infection at the mucosal surface, where they may (i) contribute to immediate protection against microbes, (ii) augment induction of adaptive immunity, and (iii) potentially provide immunological memory.

## MAIT Cells at a Glance

*TRAV1-2* expressing T cells were originally described in 1993 by Porcelli et al. as a population of TCRαβ T cells, enriched in the CD4^−^CD8^−^ (double negative) T cell subset of human blood, expressing the invariant TCRα chain *TRAV1-2* paired with *TRAJ33* (Vα7.2J33) (12). The authors suggested that these invariant TCR sequences were indicative of restriction by a non-polymorphic MHC molecule potentially presenting a limited family of antigens. Tilloy et al. further described this population of *TRAV1-2* expressing T cells as a TAP-independent and β2-microglobulin-dependent T cell subset (13). A decade after their original description, Treiner et al. described the non-polymorphic non-classical MHC molecule, MR1, as the antigen-presenting molecule for *TRAV1-2/TRAJ33*-expressing T cells (1). Furthermore, they described these cells as being enriched at mucosal tissues, including the small intestine, and thereby named them MAIT T cells.

Within this seminal study, Treiner et al. presented data demonstrating for the first time that there was a relationship between MAIT cells and microbes. MAIT cells were found to be absent in germ-free mice, indicating their expansion in the periphery depended on microbial ligands (1). In 2010, MAIT cells were shown to have a physiological role in their capacity to detect bacteria and fungi and secrete pro-inflammatory cytokines (7, 8). Consistent with this finding was the discovery of the first set of MR1 ligands, which were identified as small molecule vitamin B metabolites derived from microbes (6). Cumulatively, these studies suggested that MAIT cells could serve as innate-like T cells in the antimicrobial immune response. This hypothesis was supported by mouse models where deletion of MR1, and hence MAIT cells, rendered mice more susceptible to bacterial infections (9–11).

## MAIT Cells are Antimicrobial

In 2003, Treiner et al. found that MAIT cells were absent in germ-free mice; yet, they could be reconstituted by the oral inoculation of single bacterial species, we now know to be MAIT-activating, including *Enterobacter cloacae* or *Lactobacilius acidophilus* (1, 8). By contrast, iNKT cell frequencies were unaltered in germ=free mice (14). These data suggested that MAIT cells, but not iNKT cells, required microbial ligands for expansion in blood and tissue. A decade later, two simultaneous studies presented definitive evidence that MAIT cells were reactive to antigens produced by bacteria and fungi presented by MR1(7, 8).

The first study was predicated on the observation that a significant proportion of CD8^+^ T cells from the blood of humans who had no prior exposure to Mtb could produce IFN-γ when co-cultured overnight with dendritic cells (DCs) infected with Mtb (15, 16). Given that responses to known protein ligands were limited to those individuals with evidence of previous infection with Mtb, we postulated that these responses could either reflect previous exposure to antigens derived from ubiquitous environmental mycobacteria or could be non-classically restricted T cells.

Direct demonstration of the presence of mycobacteria-reactive, non-classically restricted CD8^+^ T cells came from a study of human thymocytes, a population of antigen-inexperienced T cells (4). In this work, Mtb-reactive CD4^−^ thymocytes with the ability to produce IFN-γ directly *ex vivo* were readily detected in all donors tested. Furthermore, the functional capacity of these cells was not altered in the presence of W6/32, a pan-HLA (HLA-A, B, C) blocking antibody, suggesting that these thymocytes were restricted by a non-classical MHC molecule.

In an effort to characterize the MHC restriction of human Mtb-reactive CD8^+^ T cells, limiting dilution analysis (LDA) cloning was used to isolate CD8^+^ T cell clones from the blood of either uninfected individuals or individuals infected with Mtb (15). The majority (64%) of the Mtb-reactive CD8^+^ T cell clones generated from subjects with active TB were classically restricted T cells, as they responded uniquely to antigen-presenting cells (APCs) from HLA-matched donors but not those that were HLA-mismatched. By contrast, 85% of the CD8^+^ T cell clones generated from uninfected individuals with no previous exposure to Mtb detected antigen that was non-classically restricted, consistent with their ability to respond to HLA-mismatched APCs. Expansion of non-classically restricted T cells from all donors allowed for more detailed analysis of the restricting allele. While the addition of either W6/32 or anti-CD1 antibodies did not result in diminished T cell activation, the addition of an anti-MR1 antibody resulted in complete inhibition. Furthermore, these MR1-restricted clones expressed the invariant TCRα chain, TRAV1-2, consistent with their characterization as MAIT cells (7). In addition to Mtb, the MAIT cell clones could be activated by DCs infected with bacteria and fungi, including *Salmonella enterica* serovar *typhimurium*, *Escherichia coli*, *Staphylococcus aureus*, *Candida albicans*, and *Sacchromyces cerevisiae* in an MR1-dependent manner. Taken together, these data indicated that MAIT cells were present in humans with no previous exposure to Mtb, and that these cells recognized cells infected with bacteria and fungi.

In a parallel study, Le Bourhis et al. also showed that purified CD3^+^ TRAV1-2^+^ CD161^+^ MAIT cells from humans could be activated by monocytes infected with *E. coli* or *Mycobacterium abscessus* in an MR1-dependent manner (8). Using TCR-transgenic mice expressing *TRAV1 TRBV6*-transgenic T cells, they demonstrated that MAIT cells were activated by DCs infected with multiple bacterial and fungal species, including *E. coli*, *Pseudomonas aeruginosa*, *Klebsiella pneumonia*, *L. acidophilus*, *S. aureus*, and *S. epidermidis*, *C. albicans*, *C. glabrata*, and *S. cerevisiae*. Importantly, these studies suggested that the bacteria *Listeria monocytogenes, Streptococcus group A*, and *Escherichia faecalis*, and as shown in both studies, all viruses tested, were not able to activate MAIT cells (7). For the first time, these studies demonstrated that specific microbes activated MAIT cells in an MR1-dependent manner and proposed a role for MAIT cells in antimicrobial immunity.

## MAIT Cell Development

Mucosal associated invariant T cells, like other TCRαβ T cells, depend on the thymus for their development (1–3). In the thymus, MAIT cells undergo TCR rearrangement through somatic recombination followed by positive selection (1–3). However, while classically restricted T cells are positively selected on thymic epithelial cells expressing MHC-I or MHC-II, murine MAIT cells are selected on CD4^+^CD8^+^ double positive hematopoietic cells that express high levels of MR1(2, 3). In humans, MR1 is also expressed on human hematopoietic CD4^+^CD8^+^ thymocytes, suggesting human MAIT cells are selected in a similar manner to mouse MAIT cells (5). Unlike classically restricted thymocytes, human MAIT thymocytes produce TNF and IFN-γ in response to bacterially infected cells (4, 5). This thymically acquired functional capacity is distinct from that of naïve classically restricted T cells that require antigen-dependent activation in the periphery followed by division and differentiation, a process that delays acquisition of effector function.

While thymic MAIT cells have the functional capacity to serve as “innate” effector T cells, their cell surface phenotype is similar to that of a naïve T cell. Following thymic egress, MAIT cells in the periphery expand and acquire a cell surface memory phenotype indicative of antigen-experience (7). To this extent, human CD8^+^ TRAV1-2^+^ MAIT cells appear to be enriched within the CD8^+^ T cell subset of the blood compared to the thymus (4, 5, 17). These data imply that while MAIT cells gain effector capacity in the thymus, they proliferate and expand in the periphery, presumably due to exposure to exogenous antigen.

## Human MAIT Cell Phenotype in Peripheral Blood and Tissues

Mucosal associated invariant T cells egress from the thymus into the blood where they circulate the lymphatic system and acquire an effector memory phenotype, indicated by cell surface expression of CD45RA^−^CD45RO^+^CD95^hi^CCR7^−^CD62L^lo^ (5, 18). MAIT cells are abundant in human blood and in a number of tissues, including the small intestine, lungs, and liver (1, 7, 8, 18–21). Indeed, MAIT cells received the name “mucosal-associated invariant T cells” based on early data suggesting their enrichment in the lamina propria of the small intestine (1). Although their frequencies and role at mucosal tissues are largely unknown, these data indicate that MAIT cells accumulate at sites where foreign microbes have the potential to gain access to the body.

Human MAIT cells were originally defined as double negative or CD8^+^ T cells that expressed *TRAV1-2* mRNA transcripts by PCR (12). However, given that classically restricted T cells and CD4^+^ CD1b-restricted germline-encoded mycolyl lipid (GEM) T cells also express TRAV1-2, TRAV1-2 expression alone is not sufficient for the definition of a MAIT cell. MAIT cells can also be characterized by high expression of the C-type lectin receptor, CD161 (KLRB1) (2). Although the physiological function of CD161 is still not known, a recent study found that TCR-dependent MAIT cell activation could be blocked by anti-CD161 antibodies (22). Currently, the co-expression of CD161 and TRAV1-2 has been widely used to delineate MAIT cells *ex vivo* (2).

TRAV1-2^+^CD161^+^ MAIT cells have been associated with additional cell surface markers, including the IL-18R, the dipeptidyl peptidase-4 or CD26, the ABCB1 drug resistance transporter (CD243), and the chemokine receptors CCR6, CXCR6, and CCR5, which are associated with trafficking to tissues including the intestine and liver (2, 8, 18, 23). Furthermore, the transcription factor PLZF (ZBTB16), which was previously associated with “innate” effector function in CD1d-restricted iNKT cells in mice, has been associated with MAIT cells in the thymus, blood, and tissues of humans as early as the second trimester of gestation (2, 21, 24).

Following the discovery that MAIT cells were activated by microbial ligands, MAIT cells have been identified in several ways including function. Functional MAIT cells have been defined as CD8^+^ TRAV1-2^+^ T cells that produce the pro-inflammatory cytokines, IFN-γ and TNF, in an MR1-dependent manner when co-incubated with infected cells (6–8, 25). To overcome the limitation that CD161 down-regulation can occur in activated MAIT cells, a simple phenotypic panel for defining those MAIT cells with the capacity to detect infected cells in the absence of *ex vivo* stimulation was identified for MAIT cells from healthy human blood. Sharma et al. demonstrated that all CD8^+^ TRAV1-2^+^ CD26^hi^ T cells could produce TNF in response to infected cells (25). MAIT cells have also been reported to produce IL-17 in response to TCR-independent stimulation (18). Therefore, while peripheral blood MAIT cells with pro-inflammatory function can be defined phenotypically as CD8^+^ or double negative TRAV1-2^+^ CD26^hi^ CD161^hi^ T cells, the phenotype of tissue=derived MAIT cells, or potentially functionally distinct MAIT cell subsets, remains to be validated.

The elucidation of the crystal structure and identification of small molecule ligands for MR1 have resulted in the generation of a human MR1 tetramer (6, 19). The MR1 tetramer consists of four molecules of biotinylated MR1, each presenting one MAIT cell antigen (rRL-6-CH2OH or 5-OP-RU), and bound to four molecules of fluorescently labeled streptavidin. In human blood and small intestine, this tetramer identifies MAIT cells, as it binds to virtually all TRAV1-2^+^ CD161^hi^ T cells in a TCR-dependent fashion (19). Using the human MR1 tetramer, a number of novel observations have been made (19, 26). For example, Rentragoon et al. have shown that *TRAV1-2* can rearrange with *TRAJ12* and *TRAJ20* in addition to *TRAJ33*, suggesting MAIT cell TCRs are more diverse than originally thought (19). In addition, their data suggest that a subset of CD3^+^ T cells binding the MR1 tetramer have minimal to no expression of CD161. These data may indicate that CD161 is not sufficient for MAIT cell identification. Thus, the MR1 tetramer will allow for detection of MAIT cells regardless of their *ex vivo* cell surface molecule expression or anti-microbial function, facilitating an unbiased characterization of MAIT cells in human health and disease.

## MAIT Cells in Mice

Mouse MAIT cells, defined by expression of the invariant TCRα chain *TRAV1 TRAJ33* (Vα19Jα33), are present at very low frequencies in wild-type C57BL/6 and BALB/c laboratory mouse strains (1). Identification of MAIT cells in mice has been significantly hindered by the lack of a commercially available antibody for the murine TCRα chain, *TRAV1*. NK1.1 was shown to be expressed on mouse MAIT cells derived from transgenic C57BL/6 mice (27). However, NK1.1 is expressed on a limited number of laboratory mouse strains, and therefore its utility in defining MAIT cells is not clear. Due to the reagent limitations and the relatively low frequency of MAIT cells in mice, their identification has been restricted to the isolation of double negative T cells via fluorescence-activated cell sorting (FACS), followed by the quantification of *TRAV1 TRAJ33* transcripts (1). Nonetheless, several *in vivo* mouse studies have contributed valuable insight suggesting a role for MAIT cells in the early immune response to respiratory pathogens (10, 11). These studies will be discussed in detail below.

## MAIT Cells have Considerable TCR Diversity

While little is known about the TCR repertoire of MAIT cells in the thymus, their repertoire in peripheral blood has been the subject of several recent reports. These studies have shown that the MAIT cell TCR repertoire in the periphery is more heterogeneous than previously thought (19, 28, 29). Historically, MAIT cells were defined by their expression of the TCRα chain *TRAV1-2* adjoined to *TRAJ33* and paired with a limited set of TCRβ chains (*TRBV6* and *TRBV20*) (13). This definition was further refined based upon the finding of *N*-nucleotide additions within the Vα and Jα (CDR3α) junctional region, lending to the modification of their name from “invariant” to “semi-invariant” (26, 30). This limited array of TCRα and β chains suggested MAIT cells detected a conserved antigen. However, more recently, several groups have identified substantial diversity in TRAJ gene usage, TCRβ chain pairing, and the CDR3α regions associated with *TRAV1-2* of MR1-restricted T cells (19, 28). Heterogeneity in TCR usage suggests the possibility that MAIT cells recognize a more diverse set of ligands.

## MAIT Cells Recognize Microbe-Derived Riboflavin Metabolites

Recently, the laboratories of McCluskey and Rossjohn successfully identified small molecules derived from the folic acid (vitamin B_9_) and riboflavin (vitamin B_2_) metabolic pathways as the first known ligands for MR1(6). They determined that metabolites from the riboflavin pathway, but not the folic acid pathway, activated Jurkat T cells expressing the invariant TCRα chain *TRAV1-2* paired with *TRBV6.1*, *TRBV6.4*, or *TRBV20*. These data were intriguing as previously the only known antigens for T cells consisted of peptides, glycolipids, sphingolipids, and in mice short formylated peptides (31). Thus, these data identified small molecules as a new and unique class of T cell antigens.

Using culture supernatant from *Salmonella typhimurium*, a microbe previously shown to activate MAIT cells, the ribityllumazines, 6-hydroxymethyl-8-d-ribityllumazine (rRL-6- CH_2_OH), 7-hydroxy-6-methyl-8-d-ribityllumazine (RL-6-Me-7-OH), and 6,7-dimethyl-8-d-ribityllumazine (RL-6,7-diMe) were found to bind MR1 and activate MAIT cells. Furthermore, the molecule rRL-6-CH_2_OH proved to be the most efficient at activating human MAIT cells. These data suggest that there are multiple ligands for MR1 that may vary in their ability to activate the MAIT TCR. In addition to ribityllumazines, a photo degradation product of folic acid, 6-formyl pterin (6-FP), was identified, bound to MR1’s ligand binding cavity in the first crystal structures (6, 32). However, unlike riboflavin metabolites, MR1-bound 6-FP was unable to activate MAIT cells. Given these data, it is possible that molecules derived from other metabolic pathways in addition to riboflavin and folic acid might provide additional sources of MR1 ligands. Interestingly, although both microbes and plants can produce folic acid and riboflavin, only ribityllumazines have been shown to activate MAIT cells. It remains to be determined if these exogenously produced molecules represent the complete repertoire of MR1 ligands.

Recent studies have characterized additional MR1 ligands, termed “neo-antigens” (33). Neo-antigens are small organic molecules, generated by the modification of unstable riboflavin metabolites by glyoxal/methylgyoxal, and are identified as 5-(2-oxoethylideneamino)-6-d-ribitylaminouracil(5-OE-RU) and 5-(2-oxopropylideneamino)-6-d-ribitylaminouracil(5-OP-RU). These neo-antigens can bind MR1 and activate MAIT cells, implying that in addition to the previously defined riboflavin intermediates, molecular bi-products created from the interaction between microbial- and potentially host-derived small organic molecules may represent new antigens for MAIT cells. It remains possible that riboflavin metabolites and their physiological bi-products differ between microbial species, providing a plausible basis for MAIT cells to respond selectively to discrete microbes. In support of this hypothesis, Gold et al., have found selective microbe-associated TCR usage within an individual. In brief, functional MAIT cells were isolated from four human donors based on their response to *Mycobacterium smegmatis*, *S. typhimurium*, or *C. albicans* (28). Evaluation of the MAIT cell TCR repertoire of each individual demonstrated that there are distinct TCR usages in response to the three different microbes. These data suggest that MAIT cells could be selectively expanded in response to distinct microbial ligands. The possibility that TCR usage could be associated with selective ligand recognition was confirmed by the finding that individual MAIT cell clones had distinctive functional responses to two known ribityllumazine antigens. MAIT cells may nevertheless expand in the periphery due to ongoing exposure to exogenous antigen that may or may not be associated with microbial infection. In sum, these data suggest that individual MAIT TCRs can display ligand selectivity, implying that, like classically restricted T cells, an individual’s MAIT TCR repertoire may reflect previous or ongoing microbial exposures.

While most microbes produce riboflavin, the process and molecules involved in the metabolic pathway are markedly different between bacterial and yeast species (34). Some microbes such as *M. smegmatis* and several *Candida* species are considered “overproducers” of riboflavin (34). Thus, the ability of microbes to produce riboflavin has been reported to fluctuate dramatically depending on the availability of chemical elements, including, but not limited to, iron, manganese, zinc, and magnesium (34). These data indicate that the physiological levels of these molecules may affect antigen production by the microbe. However, the mechanisms by which many microbes individually regulate riboflavin synthesis and how chemical elements may regulate these pathways remain unknown. As a result, it remains an open question as to whether or not MAIT cells with pathogen specificity can be defined. Thus, one of the top priorities in the field of MAIT cell immunobiology is the identification of these microbial antigens.

## MR1 Structure

**Figure 1:**
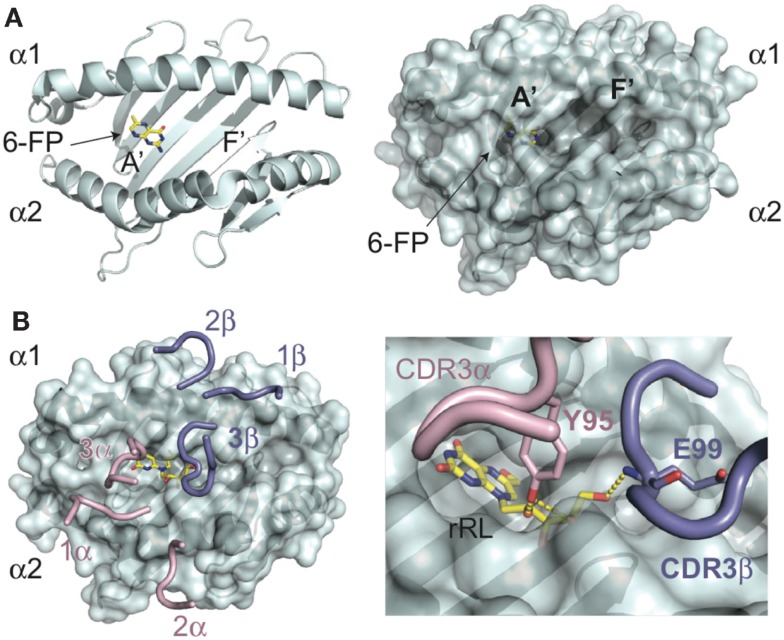
**Molecular basis of antigen presentation by MR1 and recognition by MAIT TCRs**. **(A)** Backbone (ribbon) and surface representations of the structure of human MR1 (PDB ID: 4GUP) are shown in cyan. In the MR1, A′ pocket is shown the 6-FP ligand in yellow, with nitrogen atoms colored blue, and oxygen in red. The A′ and F′ pockets are labeled as such. **(B)** MAIT TCR recognition of MR1 presenting the stimulatory antigen, rRL (rRL-6-CH_2_OH) (PDB ID: 4LCC), only CDR loops of the MAIT TCR are shown, positioned in complex with MR1. CDRa loops are colored pink; CDRb are shown in blue. The rRL antigen is shown as above for 6-FP. In the insert is a zoomed view of the CDR3a (Y95) and CDR3b (E99) loop interactions with the ribtyl chain of rRL, showing the hydrogen-bonds as yellow dashes.

The structural elucidation of MR1 provided the first insight into the nature of ligands presented by this molecule (6, 32). The overall backbone structure of MR1 is most closely related to the classical class I MHC, HLA-A2; however, the antigen-presenting groove has a number of unique features. The MR1 cavity is smaller and has two pocket structures capable of binding small molecule antigens (Figure 1A). The A′ pocket, named as such due to the similarity in location to the A′ tunnels in CD1 molecules, is lined with aromatic and basic residues, creating a small, positively charged cavity that is almost entirely sequestered from external solvent. An additional pocket, termed F′ (similar in location to the F′ tunnel in CD1 molecules), is more shallow and can be variable in size due to flexibility noted in the structure of the α2 helix of human MR1(6). A comparison between the two molecules in the asymmetric unit of the human structure revealed an ~11Å shift between the positioning of the N-terminal portion of the α2 helix, with one molecule having a significantly inward-shifted helix resulting in a pseudo-collapsed F′ pocket. The structure of the A′ pocket was almost identical between the two structures, suggesting that this flexibility would not directly affect antigens presented in this pocket, although this phenomenon may shed light onto the molecular mechanisms of ligand loading. Curiously, this conformational flexibility was not apparent in the structure of bovine MR1 solved in complex with a MAIT TCR; either bovine MR1 has greater rigidity in this region or complexation with a TCR stabilizes this region of MR1(32).

In both crystal structures of MR1, electron density consistent with a bound ligand in the A′ pocket was apparent. The protein used in producing these crystals came from two different sources; human MR1 was refolded in the presence of RPMI culture media, whereas bovine MR1 was secreted from insect cells. The ligand from human MR1 was characterized as 6-FP, a pterin-based compound discussed previously. The electron density in bovine MR1 was similar to that identified in human MR1, consistent with a pterin-like compound such as 6-FP. This compound is prevalent in cell culture media, explaining its availability for forming complexes with MR1. In both structures, the ligand electron density was continuous with a lysine residue (K43) deep within the A′ pocket, supporting a covalent attachment of this compound via Schiff base. While it has yet to be demonstrated whether 6-FP and the covalent attachment are relevant in the normal functioning of MR1 presentation *in vivo*, exogenous addition of this compound leads to up-regulation of MR1 on the cell surface, yet does not lead to, in fact can be antagonistic toward MAIT activation (6), suggesting it can stabilize MR1 but cannot provide a stimulatory signal to MAIT cells through their TCR.

The elucidation of lumazine-based compounds, previously described as MAIT cell antigens, is variants of ribityl-lumazine compounds, which contain a two-ring core structure with a ribityl extension. The ring structures are well-accommodated within the aromatic environment of the A′ pocket and, depending on the lumazine derivative, also establish hydrogen-bonding networks with the basic residues located within this pocket. The ribityl group extends from the A′ pocket and is engaged to differing degrees depending on the orientation of the lumazine group, by the MAIT TCR (30, 35). It is these additional ligand-mediated contacts between the CDR3α and, depending on the sequence of the MAIT TCR, CDR3β loops that enhance MAIT TCR binding leading to MAIT cell activation (Figure 1B). Other derivatives of intermediaries of the riboflavin pathway that are highly stimulatory to MAIT cells, including the “neo-antigens” 5-OE-RU and 5-OP-RU, are presented by MR1 via a covalent Schiff base attachment (33). All characterized ligands for MR1 have been shown to be presented through interactions, either covalent or non-covalent, with residues lining the A′ pocket. Whether there are other ligands for MR1, potentially being presented also in the F′ pocket, is unclear at this time, although considering the universe of ligand possibilities for this protein it is likely that there are at least some non-B vitamin small molecules that derive from endogenous or exogenous sources that can be presented by MR1. Indeed, it is likely the B vitamin ligands identified thus far are only a subset of the ligand repertoire presented by this intriguing MHC-like protein and that there are other small molecule candidates for activating MAIT cells during microbial surveillance and, potentially, other non-microbial related surveillance activities.

## MR1-Dependent Antigen Processing and Presentation

MR1 transcripts and protein are expressed ubiquitously in all mammalian cells tested (36–38). However, unlike classical MHC molecules, the MR1 protein resides primarily in intracellular endocytic compartments, while cell surface expression is often undetectable (39). One hypothesis is that MR1 is under tight regulation as to avoid indiscriminate activation of MAIT cells. To this extent, cells infected with Mtb, a pathogen known to elicit MAIT cell responses, or cells treated with the newly identified MR1 ligand, 6-FP, increased and stabilized expression of MR1 on the cell surface (5, 40). These data suggest that the availability MR1 ligand(s) is requisite for MR1 surface expression and therefore MAIT cell activation.

A recent study implies that in humans with dysregulated immune systems, MAIT cell prevalence and activation were associated with pathology (41). For example, MAIT cells were enriched and activated, as determined by expression of Ki67, NKG2D, and BTLA, in ileum tissue sections taken from humans with inflammatory bowel disease compared to healthy control subjects (41). Thus, careful regulation of MR1 expression may be necessary in tissues such as the gut where exposure to microbes is ubiquitous. Regulation of surface expression of MR1 appears to be a limiting factor for MAIT cell activation, as stabilizing endogenous MR1 cell surface expression *ex vivo* has the potential to activate MAIT cells (42). At present, the mechanisms by which antigens are processed and presented in the context of MR1 are largely unknown.

## MAIT Cells Play a Protective Role in Antimicrobial Immunity

Le Bourhis et al. provided the first *in vivo* evidence suggesting that MAIT cells were protective against bacterial pathogens (8). In this study, *TRAV1* (iVα19) and *TRBV6* (Vβ6)-transgenic mice, on an MR1-sufficient (MR1^+/+)^ or an MR1-deficient (MR1^−/−^) background, the latter of which lack MAIT cells, were injected intraperitoneally with *E. coli* or *Mycobacterium absessus*. In both cases, MR1^−/−^ mice had increased bacterial burden implicating a role for MAIT cells in the protection against two different microbes. In this same study, control C57BL/6 mice expressing wild type levels of MR1 had no significant change in mycobacterial burden in comparison to MR1^−/−^ mice. Notably, *TRAV1*- and *TRBV6*-transgenic mice have increased numbers of MAIT cells in comparison to wild type mice, indicating that the augmented frequencies of MAIT cells in the transgenic mice resulted in protection (2). Following this study, several groups have found that MAIT cells were associated with early protection against bacterial pathogens in mouse models of infection, the details of which will be discussed below.

## Mycobacteria

In an effort to determine the role of MAIT cells in protection against mycobacterial infections *in vivo*, Chua et al. challenged MR1^−/−^ mice with a low dose aerosol infection of *Mycobacterium bovis* BCG (10). MR1^−/−^ mice had significantly increased bacterial burden at day 10 following infection in comparison to MR1^+/+^ mice. However, at day 30 post-infection, the bacterial burden of MR1^+/+^ and MR1^−/−^ mice were not significantly different. These data suggest that MAIT cells enhance early containment of mycobacterial infection in the lungs. Further studies are needed to determine if MR1^−/−^ mice infected with aerosolized Mtb will recapitulate this phenotype.

In the same study, MAIT cells contributed to enhanced bacterial containment within macrophages in an MR1-independent manner. Survival of BCG in macrophages was dramatically reduced during an *in vitro* co-culture of enriched MAIT cells, derived from the double negative, CD8^+^, and CD4^+^ T cell subsets of *TRAV1* transgenic mice. However, when a similar fraction of cells from wild type mice (which contain very few MAIT cells) were co-incubated with infected macrophages, no effect on bacterial containment was noted. To determine if the anti-mycobacterial capacity of MAIT cells was dependent on activation through MR1, MAIT cells were co-incubated with MR1 deficient macrophages that were infected with BCG. Surprisingly, absence of MR1 did not preclude MAIT cells from reducing intracellular survival of BCG. To determine if IL-12 secretion by infected macrophages was responsible for MAIT cell activity, a blocking antibody to IL-12 was included in a co-incubation of MAIT cells and infected macrophages. This experiment showed that in the absence of IL-12, MAIT cells lost their bactericidal capacity. The authors suggest that MAIT cell activation and antibacterial capacity were dependent on IL-12 and not MR1. However, the contribution of IL-12 *in vivo* and the mechanism by which it enhanced mycobacterial control were not directly demonstrated.

The possibility that MAIT cells can be activated in an MR1-independent manner has been supported by the recent observation that IL-12 and IL-18 production by macrophages infected with *E. coli* is sufficient to induce IFN-γ production by human liver-derived MAIT cells following an overnight co-culture (20). Given the ability of IFN-γ to augment IL-12 production, it is possible that IL-12 secretion could stimulate a positive feedback cycle in which MAIT cell production of IFN-γ further amplifies the production of IL-12, which in turn promotes further MAIT cell activation. By contrast, the ability of human blood-derived MAIT cells to produce IFN-γ in response to mycobacteria-infected APCs has been shown to be MR1-dependent (7, 8).

## Klebsiella

Georgel et al. evaluated the role of MAIT cells in MR1^−/−^ mice given a high dose (2 × 10^8^/mouse) intraperitoneal infection with the Gram-negative bacterium, *K. pneumonia* (9). In wild type mice, *K. pneumonia* was cleared within the first 72 h of infection, whereas in MR1^−/−^ mice, bacteria had spread from the site of injection to several tissues including the lungs. Overall, the MR1^−/−^ mice had significantly increased mortality. These data are in agreement with that obtained in the BCG challenge model, in that mice lacking MAIT cells had delayed bacterial clearance, suggesting MAIT cells facilitate the early recognition and clearance of bacterial infection.

In the same model, MR1^−/−^ mice showed equivalent protection to wild type mice when infected with other Gram-negative species, including *E. coli*, S*higella dysenteriae*, and *Yersinia enterocolitica*. These data would suggest that MAIT cells are more important for clearance of some bacterial pathogens but not others. In this regard, it is important to note in this study each of the bacterial strains were administered by intraperitoneal injection, and therefore it remains possible that MAIT cells could facilitate bacterial containment of the same strains following mucosal routes of infection. Here, we postulate that MAIT cells may have anatomic localization that might favor their role in the respiratory tract.

## Francisella

In support of the hypothesis, Meierovics et al. evaluated the role of MAIT cells in the host response to a low-dose intranasal infection with the Gram-negative bacterium, live vaccine strain of *Francisella tularensis* (LVS). In wild type mice, MAIT cells were enriched in the lungs as early as 8 days and up until the latest time point tested (18 days) post-infection. Furthermore, in wild type mice, the frequency of MAIT cells increased in the lung as the bacterial burden decreased, indicating an inverse correlation between the frequency of MAIT cells and the bacterial burden. Alternatively, at days 10–15 post infection, MR1^−/−^ mice had significantly higher bacterial burden, 1 × 10^6^ CFU versus 1–5 × 10^5^ CFU in wild type mice. Whereas it took wild type mice ~17 days to clear the infection, it took MR1^−/−^ mice five additional days (~22 days). These findings support the hypothesis that MAIT cells contribute to early control of LVS in mice.

Mucosal associated invariant cells in the lungs of mice infected with LVS were pro-inflammatory as they produced IFN-γ, TNF, and IL17A during both early and late infection. As described for the intracellular containment of BCG, co-incubation of purified MAIT cells with LVS-infected macrophages resulted in bacterial growth inhibition in an IL-12-dependent manner. Together, these data suggest that the increase in MAIT cell frequencies in response to pulmonary LVS infections and early control of the bacteria within the lungs is MR1-dependent, but that IL-12 plays a direct role in the containment of intracellular infection.

## A Role for MAIT Cells in Human Antibacterial Immunity

Several studies have implicated MAIT cells in the recognition and possible control of intracellular infection in humans. MAIT cells are present in equal or greater numbers in the lungs of healthy humans, compared to peripheral blood and lymph nodes (7, 8). Thus, MAIT cells are physically positioned to serve as sentinels in the detection of respiratory pathogens, such as Mtb, in the lung. The plausibility of MAIT cells serving as sensors of pulmonary infection with Mtb is supported the observation that subjects with pulmonary TB have diminished MAIT cell frequencies in the peripheral circulation (7, 8). These findings could reflect a dynamic relationship where the number of MAIT cells in peripheral blood is inversely correlated with the presence of Mtb in the lung. Here, we postulate that the presence of mycobacterial antigen in the lungs results in trafficking to the site of infection and possible expansion of antigen-selective MAIT cells. The hypothesis that bacterial infection could result in the egress of MAIT cells from the peripheral circulation is supported by the observation that MAIT cell frequencies are diminished in the blood of individuals with bacterial sepsis (43).

**Figure 2:**
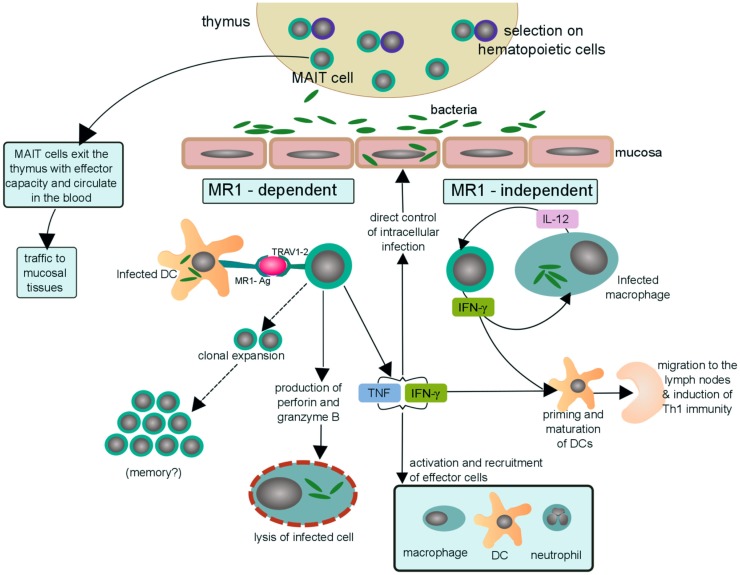
**Possible mechanisms of MAIT cells in human antibacterial immunity**. MAIT cells with effector function egress from the thymus and circulate through the blood and lymph as well as traffic to mucosal tissues, including the lungs. Upon bacterial infection, a dendritic cell (or other MR1-expressing cell) can activate MAIT cells by presentation of bacterial-derived antigens on MR1 (MR1-Ag) to the MAIT TCR (TRAV1-2). MAIT cells activated in an MR1-dependent manner may then (i) undergo clonal expansion of antigen-specific MAIT cells in the lungs and thus aquire long-term memory to specific pathogens, a hypothesis that still needs to be tested. (ii) directly lyse cells infected with Mtb by secreting the cytotoxic molecules granzyme and perforin (22, 25, 44), (iii) indirectly kill mycobacteria by secreting the pro-inflammatory cytokines, TNF and IFN-γ, through recruitment and activation of effector cells to the site of infection. MAIT cells may also be activated, in an MR1-independent manner, by IL-12 produced by a local infected macrophage. The activated MAIT cell may then secrete IFN-γ and stimulate a positive feedback cycle in which IFN-γ augments expression of IL-12 by infected macrophages and vice versa. Furthermore, the abundance of IFN-γ may then contribute to priming and maturation of naïve DCs that traffic to the lymph node and initiate Th1 immunity. Thus, suggesting a possible scenario by which MAIT cells may bridge innate and adaptive immunity.

The observations made thus far in humans and mice can be used to develop a model by which MAIT cells contribute to the control of intracellular infection. This model is presented in Figure 2 and possesses the following: (1) thymically derived MAIT cells have inherent effector capacity, and by virtue of their preferential residence in mucosal sites can serve as early sensors of bacterial or fungal infection; (2) early recognition of an infected cell could result in the direct control of intracellular infection through the release of pro-inflammatory cytokines and or cytotoxic molecules; (3) early recognition of an infected cell could promote the acquisition of Th1 immunity through the release of IFN-γ which in turn promotes the production of IL-12 from tissue-resident DCs; (4) IL-12 may play a special role both in the promotion of Th1 immunity and augmentation of the MAIT cell response; and (5) microbial exposure results in the expansion and maintenance of MAIT cells that are selective for these pathogens.

## Outstanding Questions

### MAIT cells in autoimmunity

Mucosal associated invariant cells have been implicated in autoimmunity and inflammatory disorders, including chronic inflammatory demyelinating polyneuropathy, experimental autoimmune encephalomyelitis, multiple sclerosis, arthritis, celiac disease, and as mentioned previously IBD (41, 45–50). One hypothesis is that these autoimmune conditions could be instigated or exacerbated by prior or ongoing microbial infections. Here, we postulate that bacterially-derived MR1 ligands could serve as “molecular mimics” of host-derived compounds allowing for MAIT cell autoreactivity. In this context, the MAIT cell TCR may recognize host-derived pterins or pterin-like small molecules that may serve as ligands for MR1. Additionally, host-derived molecules may bind MR1 and activate MAIT cells, or like 6FP, serve to stabilize MR1.

It is also possible that MAIT cells may be activated in an MR1-independent manner by pro-inflammatory cytokines as a result of ongoing tissue inflammation. These activated MAIT cells may then secrete pro-inflammatory cytokines or exert their cytolytic potential in an indiscriminate manner. While MAIT cells have been associated with autoimmunity, it remains unknown if they serve a pathological role in these diseases. It is also possible that they play a protective or regulatory role. With the advent of the MR1 tetramer and the development of comprehensive *ex vivo* functional assessment, the physiological function of these cells in autoimmunity can be further elucidated.

### The role of MAIT cells in vaccination

Vaccines serve to initiate long-term protection through induction of adaptive immunity. Currently available vaccines are often administered systemically, and hence may be less effective in the initiation and maintenance of mucosal immunity. The desirability of mucosal vaccination might be particularly evident in infections that are spread via aerosol (51). In this regard, the role that MAIT cells might play in a mucosal vaccine is largely unexplored. One possibility is that MAIT cell expansions in the lung do not reflect long-term memory but depend on the ongoing presence of microbial ligand. In this regard, it might be envisioned that MAIT cells could be harnessed as “adjuvants” in mucosally delivered vaccines. Alternately, if MAIT cells have memory, then vaccination with selective ligands could result in the stable expansion of MAIT cells.

### Could MAIT cells be used as mucosal adjuvants?

Given that conventional systemic vaccines rely on the presence of an adjuvant to elicit sustained B cell and T cell responses, we postulate that the activation of MAIT cells in the lung could serve to facilitate the acquisition of adaptive immunity. At present, FDA-approved adjuvants are TLR agonists. While these are traditionally thought to result in the direct activation of APCs such as DCs, we postulate that the activation of lung-resident MAIT cells could promote the acquisition of Th1 immunity, and hence could serve as an adjuvant. With the recent discovery of vitamin B metabolites serving as MAIT cell antigens, one could envision ribityllumazines as a class of “Vita-PAMPS,” that if safely delivered to the mucosal surface where MAIT cells are present, could serve as a mucosal adjuvant (52).

### Could MAIT cells be targeted for a mucosal vaccine?

Alternately, we speculate that the expansion and maintenance of microbial selective MAIT cells could be used as a mucosal vaccination strategy. These approaches will depend on the identification of microbial-selective ligands, their stable formulation, and their delivery either systemically or via aerosol. While either the adjuvant or vaccination strategies are attractive, significant challenges remain. First, antigens that are selective for discrete pathogens, such as Mtb, remain to be identified. Second, many of the ligands identified to date are unstable, making them unsuitable for vaccination studies. Third, animal models that reflect a human MAIT cell immunobiology have not been developed. In this regard, a mouse model would be ideal, and will likely require the identification of mouse strains that have MAIT cell frequencies and reactivity that are similar to humans. Alternately, it is possible that other animal species, such as the rabbit, guinea pig, or non-human primates, may be needed.

## Concluding Remarks

While existing data strongly support a role for MAITs in the recognition and control of microbial infection, particularly at the mucosal surface, there remains much that is not known. For humans, a direct demonstration of the role of MAIT cells in the host response to microbial infection will either require the identification of MR1 variants that alter MAIT cell function, and potentially increase the host-vulnerability to infection, or the identification of altered developmental pathways that alter MAIT cell frequency and function. In this regard, it might be expected that these deficiencies affect other cell types, such as iNKT cells. Alternately, vaccination strategies that harness MAIT cells would also allow for more direct demonstration of the protective capacity of MAIT cells. Finally, given the tight regulation of MR1 surface expression and MAIT cell activation, it is possible that microbial activation of MAIT cells could have the unintended consequence of promoting autoimmunity.

## Conflict of Interest Statement

The authors declare that the research was conducted in the absence of any commercial or financial relationships that could be construed as a potential conflict of interest.
